# Peptide immunotherapy for childhood allergy - addressing translational challenges

**DOI:** 10.1186/2045-7022-1-13

**Published:** 2011-11-07

**Authors:** Karen J Mackenzie, Stephen M Anderton, Jürgen Schwarze

**Affiliations:** 1 MRC Centre for Inflammation Research, The University of Edinburgh, Edinburgh, UK; 2 Child Life and Health, The University of Edinburgh, Edinburgh, UK; 3 Centre for Multiple Sclerosis Research, The University of Edinburgh, Edinburgh, UK

**Keywords:** Allergy, Children, Peptide Immunotherapy

## Abstract

Allergic sensitisation usually begins early in life. The number of allergens a patient is sensitised to can increase over time and the development of additional allergic conditions is increasingly recognised. Targeting allergic disease in childhood is thus likely to be the most efficacious means of reducing the overall burden of allergic disease. Specific immunotherapy involves administering protein allergen to tolerise allergen reactive CD4^+ ^T cells, thought key in driving allergic responses. Yet specific immunotherapy risks allergic reactions including anaphylaxis as a consequence of preformed allergen-specific IgE antibodies binding to the protein, subsequent cross-linking and mast cell degranulation. CD4^+ ^T cells direct their responses to short "immunodominant" peptides within the allergen. Such peptides can be given therapeutically to induce T cell tolerance without facilitating IgE cross-linking. Peptide immunotherapy (PIT) offers attractive treatment potential for allergic disease. However, PIT has not yet been shown to be effective in children. This review discusses the immunological mechanisms implicated in PIT and briefly covers outcomes from adult PIT trials. This provides a context for discussion of the challenges for the application of PIT, both generally and more specifically in relation to children.

## Introduction

Allergic disease including atopic eczema, allergic rhinitis, allergic asthma and food allergy causes significant patient morbidity and economic costs to healthcare systems [1,2]. Current clinical management primarily relies on allergen avoidance, treating symptoms as they arise (often using medications such as β2 agonist inhalers, antihistamines and adrenaline) and generalised suppression of immune responses (e.g. using corticosteroids). Therapeutic blockade of cytokines such as interleukin-5 (IL-5) and of IgE have had varied clinical results and such approaches are reserved for highly selected patient groups at present [3-5]. Many allergic patients will first experience symptoms during childhood [6] and allergic sensitisation may begin very early in infancy, even prenatally [7,8]. Children with atopy are at risk of developing new sensitisations and additional allergic conditions as they get older [1,9]. Identifying atopic children early and modifying disease progression is therefore a hugely attractive therapeutic goal [10].

Evidence suggests a key role for CD4^+ ^T cells, particularly the T helper (Th) 2 subset, in allergy. These cells express the transcription factor GATA-binding protein 3 (GATA-3) and can produce allergy-associated cytokines such as IL-4, IL-5 and IL-13 which are implicated in a host of allergic responses such as eosinophil recruitment and airway hyperreactivity [reviewed in [11]]. Th2 cells also provide B cells with help, driving immunoglobulin class-switching towards allergen-specific IgE [11]. Less commonly, and often in concert with Th2 cells, other helper subsets such as Th1, Th17 and Th9 cells have also been implicated in the pathogenesis of allergic asthma in some patients [reviewed in [12]]. Therapeutic targeting of allergen-reactive CD4^+ ^T cells therefore has the capacity to abrogate downstream allergic responses [11]. One way of doing this is through specific immunotherapy (SIT) which targets CD4^+ ^T cells via the administration of protein allergen. First used over a century ago [13], much of SIT's therapeutic effects have been shown to result from the induction of tolerance of allergen-reactive CD4^+ ^T cells so they no longer mount an allergic response to the allergen. This can occur either through direct effects on allergen-reactive T cells and/or through the actions of T regulatory cells [14]. SIT can significantly improve symptoms in allergic patients [15], therapeutic effects can be long-lasting [16] and SIT for allergic rhinitis may reduce the likelihood of future asthma development [reviewed in [17]]. Yet SIT can also be risky. Pre-existing allergen-specific IgE can bind to multiple sites on protein allergen, leading to IgE cross-linking on mast cells, inducing mast cell degranulation and subsequent allergic reactions, even anaphylaxis [18-20].

**Figure 1:**
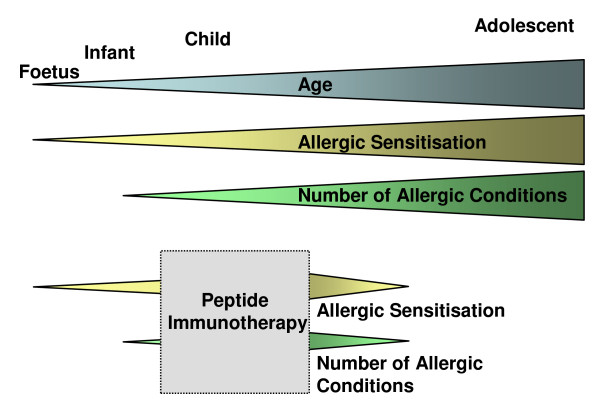
**Proposed model of the effects of PIT on disease progression in children with severe atopy**. Evidence suggests that in susceptible individuals, allergic sensitisation begins early in life, even prenatally, preceding development of allergic conditions such as eczema. The number of allergens an individual is sensitised to and the number of diagnosed allergic conditions can increase with age. Inducing tolerance to an allergen using PIT early, rather than later, in life has the potential to reduce sensitisation to additional allergens and reduce the risk of progression to multiple allergic conditions particularly for children with severe atopy.

Such SIT-associated risks can be overcome by identifying short peptides from within the protein allergen to which the CD4^+ ^T cell response is directed [10]. Such "immunodominant" peptides can bind efficiently to major histocompatibility complex II (MHC II) to induce T cell responses and can therefore be used to generate T cell tolerance, while their short length and lack of tertiary conformational structure do not facilitate IgE cross-linking [21]. Such therapeutic application of peptides [hereafter referred to as peptide immunotherapy (PIT)] was first developed in rodent autoimmune disease models [22]. However, clinical translation of PIT has been faster for allergy than for autoimmune disease [10]. The early manifestation and often progressive nature of allergic disease means that maximising PIT's disease-modifying potential would require targeting allergic children (Figure 1). However, clinical trials of PIT have so far not included children and the need for scrupulous safety concerning novel paediatric treatments necessitates further understanding of the mechanisms involved in successful PIT.

### Immunological mechanisms of PIT

PIT harnesses the body's capacity to induce peripheral T cell tolerance. This capacity is paramount to prevent inflammatory responses both to harmless exogenous antigens and to self antigens [23]. Lack of pathogen-associated or inflammation-associated "danger signals" in such circumstances promote a tolerogenic rather than an inflammatory response. Hence, administering a soluble peptide in the absence of danger signals (e.g. in the absence of lipolysaccharide (LPS) and/or an adjuvant) can induce tolerance, whereas administration of the same peptide with an adjuvant promotes an inflammatory/immunogenic response [21]. Soluble peptides administered by intranasal, oral, intravenous, subcutaneous and intradermal routes all have the potential to induce tolerance [24-27].

#### Dendritic cells

In order to elicit a T cell response, peptide must be presented to T cells by antigen presenting cells in the context of MHC II, facilitating engagement of the T cell receptor (TCR). Dendritic cells (DCs), antigen presenting cells optimally able to stimulate T cells, are strongly implicated in driving tolerance. CD11c-deficient mice (lacking conventional DCs, plasmacytoid DCs and Langerhans cells) develop spontaneous autoimmunity, implicating DCs in the maintenance of peripheral tolerance [28]. Peptide is rapidly presented on DCs after therapeutic administration [29]. It appears that the activation status of the DC is the prime orchestrator of whether or not a T cell becomes tolerant since "immature" DCs expressing low levels of costimulatory molecules have been found to induce T cell tolerance, in contrast to activated DCs [25,30]. Considering that different DC subtypes may be tissue-specific with particular anti-inflammatory or pro-inflammatory characteristics [31], the nature of DC-T cell interactions, and hence the mechanism of tolerance induction, could vary with different PIT delivery routes.

#### T cells and the 3 Pillars of Tolerance

The strength of the TCR signal induced by a peptide can affect the outcome of PIT. Peptides with strong MHC II avidity form a stable peptide-MHC II interaction, hence generate a strong TCR signal and have an improved ability to induce tolerance [32]. This may be due to a resultant rapid, and synchronous T cell response [33]. Studies, predominantly in mice, have shown that the induction of T cell tolerance is likely to be effected by three fundamental mechanisms - deletion, adaptation and regulation [34]. All have variably been thought to contribute to the effects of PIT [25-27,35,36], however, certain mechanisms may be more therapeutically desirable than others.

#### Deletion

PIT can lead to targeted deletion of CD4^+ ^T cells [25]. This mechanism is advantageous in that deletion removes the possibility of such cells reverting to pathogenicity in the future. High dose PIT and/or induction of a strong but transient TCR signal appear to favour deletion [25,37].

#### Adaptation

T cells can also be rendered tolerant through anergy - a state of unresponsiveness. A particular form of T cell anergy, known as adaptive tolerance, may be most relevant *in vivo *[38]. Adaptive tolerance is associated with inhibition of proliferation and cytokine production and has been demonstrated, to varying degrees, to occur after PIT in several murine and human studies [35,36,39-41]. One drawback is that adaptive tolerance is not necessarily permanent, and persistence of the peptide may be required to maintain tolerance [39]. Hence, adaptive tolerance alone may be insufficient to maintain long-term therapeutic effects after a short course of treatment.

#### Regulation

Several subsets of T regulatory cells capable of constraining immune responses have been described [reviewed in [42]]. Generating regulatory T cells using PIT is therapeutically desirable because of the potential for long-lived suppression of unwanted allergic responses. Using PIT to induce allergen-reactive T regulatory cells also has the potential to down-regulate responses to other allergens. This "bystander suppression" [10] could be particularly beneficial in the allergic lung where multiple potential allergens are continually encountered, and because allergic lung inflammation has been found to increase the likelihood of developing allergic sensitisation to additional aeroallergens [43].

Different T regulatory cell subsets vary in their mechanism(s) of action and whether or not they express the transcription factor Foxp3. In fact, there are limited data concerning the ability of PIT to induce Foxp3^+ ^T regulatory cells. That said, peptide-reactive Foxp3^+ ^T regulatory cells were generated in a model using a peptide complex designed to specifically target peptide to DCs, ensuring peptide uptake and presentation [44]. Interestingly, generation of Foxp3^+ ^T regulatory cells was reduced using high dose peptide or when peptide was presented by activated DCs [44].

One means whereby T regulatory cells often effect regulation is via production of immunosuppressive cytokines such as IL-10. In beekeepers, the frequency of IL-10 producing allergen-reactive T cells has been found to increase following tolerance induction to bee venom, which develops naturally in response to multiple bee stings [45]. Some murine studies of PIT have clearly demonstrated peptide-reactive T regulatory cells as the source of IL-10 following PIT [26]. IL-10 was also implicated in the therapeutic effects of PIT in a mouse model of allergic airways disease [35]. In one clinical study, IL-10 production increased from allergen-reactive T cells after PIT [36] and, in another, CD4^+ ^T cells obtained from patients after PIT had suppressive activity *in vitro *[40]. It appears that the development of regulatory mechanisms following PIT may be favoured by regimens using repeated peptide dosing [26], however the optimal dosage and delivery regimes favouring regulation remain unclear.

**Figure 2:**
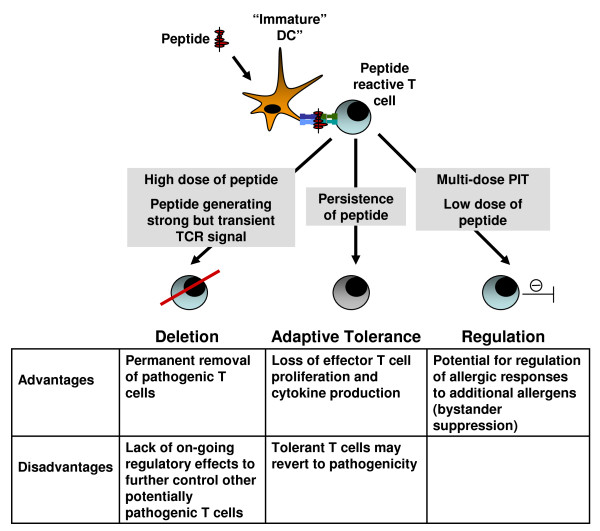
**Possible impact of the mode of peptide delivery on the mechanisms of T cell tolerance**. The nature of peptide delivery may influence the mechanism(s) of tolerance that are evoked. High dose peptide or peptides that lead to strong, transient TCR stimulation may favour deletion of allergen-reactive T cells. Persistence of peptide may favour adaptive tolerance and thus anergy/unresponsiveness of allergen-reactive T cells. Adaptive tolerance may, however, be reversed when peptide no longer persists. Regulatory mechanisms may be favoured by multi-dose regimens and/or low dose applications.

Overall, therapeutic targeting of allergen-reactive (principally Th2) CD4^+ ^T cells using PIT could, therefore, occur via deletion, adaptation, or regulation, or most probably a combination of these, echoing the varied mechanisms attributed previously to clinical effectiveness following SIT [[46,47] and reviewed in [14]]. By targeting allergen-specific CD4^+ ^T cells, and consequently modulating the provision of B cell help, PIT has also been found to be capable of beneficially altering allergen-specific antibody levels in some studies [[35] and discussed in [48]]. While deletion or adaptation of allergen-reactive Th2 cells should reduce allergic responses to that particular allergen (in part because reductions in Th2 cytokine production should inhibit the recruitment of innate immune cells such as eosinophils), the induction of regulatory mechanisms also confers the potential to suppress allergic responses to a variety of other allergens and is thus particularly advantageous. Existing data from limited human studies of PIT and from experimental murine PIT models (discussed above) indicate that the mechanism of tolerance induction that predominates following PIT is likely to be influenced by factors such as the nature of disease, the dose of peptide and the delivery route (Figure 2).

### PIT in adult allergy trials

Since the first clinical PIT trial in adult allergic patients in the 1990s [49] significant advances have been made, but as yet there are no PIT studies involving children. The majority of clinical PIT studies have utilised peptides from the major cat allergen Fel d 1. The outcomes from these trials have been comprehensively reviewed previously [24] and will therefore not be covered in detail here. Briefly, however, initial trials used two 27 amino acid immunodominant Fel d 1 peptides. In those studies, short term improvements in lung function tests and improvements in clinical symptoms were described to a variable extent [49-52]. More recently, there has been a move towards using shorter peptides, typically 15-17 amino acids in length. For Fel d 1, these were administered in the form of multiple, overlapping peptides encompassing the majority of the Fel d 1 protein. This has the advantage of maximising the number of allergen-reactive T cells that may be tolerised since patients may respond to multiple immunodominant peptides and immunodominant peptides may vary between individuals with different HLA types. Using shorter peptides also abrogated the risk of allergen-specific IgE binding which had occasionally been reported in studies using longer peptides [50]. This approach using multiple short, overlapping peptides reduced late-phase skin reactions to cat allergen, reduced proliferation of peripheral blood mononuclear cells (PBMC) to cat allergen and improved symptoms in some patients [53,54]. In one study, PBMC responses to Fel d 1 peptides not included in the treatment vaccine were also reduced, implying linked-suppression, whereby tolerance induced to one peptide inhibits responses to other peptides within the same protein [35].

PIT also offers a potentially safer approach to allergen-specific immunotherapy for bee venom allergy, where SIT has a high frequency of severe allergic reactions [55]. PIT using peptides from the phospholipase A2 bee venom allergen has led to reduced T cell proliferation to those peptides and altered cytokine profiles e.g. by increasing IL-10, in some studies [36,56,57]. PIT has also been found to reduce the severity of allergic responses to deliberate bee stings [56].

PIT can therefore improve clinical outcomes in allergic patients in some instances. Furthermore, although PIT studies have so far focused on aeroallergens or venom allergies, future application of PIT for the treatment of food allergies is also a possibility, particularly given the continued identification of food allergen derived T cell epitopes [58]. It is, however, apparent from clinical studies so far that the effects of PIT can be variable and therapeutic effects are not necessarily seen across all clinical readouts. These inconsistencies are likely due in part to inter-trial variability of factors such as clinical readouts, PIT regimens, nature of disease and whether patients have received immunotherapy previously, which can complicate the assessment of clinical effectiveness.

### Difficulties with PIT

#### Does PIT remove the risk of anaphylaxis associated with SIT?

The need to avoid severe IgE-driven hypersensitivity reactions, including anaphylaxis, is the primary reason why PIT rather than SIT may be more applicable to treating allergic children. Crucially, anaphylaxis and other severe hypersensitivity reactions are extremely rare after PIT. However, a PIT trial involving multiple sclerosis (MS) patients was halted due to a number of hypersensitivity reactions [59]. The peptide used contained sequence alterations designed to improve its therapeutic effects [an altered peptide ligand (APL), further discussed below]. Interestingly, it was levels of IgG1 and not IgE which were elevated in patients displaying hypersensitivity reactions [59]. This potential for generation of anaphylaxis-promoting peptide-specific IgG1 should be noted, although importantly this has not been reported in PIT allergy studies.

#### Long-term disease exacerbation

To our knowledge, there are no reports of PIT inducing long-term exacerbation of allergic disease. However, a phase II PIT trial using an APL in MS patients was discontinued due to disease exacerbations [60]. The APL displayed low immunogenicity and good tolerogenic potential *in vitro *yet seemed to promote an inflammatory T cell response in some patients. Exacerbation of disease has also been noted to occur using an unaltered peptide in a murine allergy model [61]. These rare examples highlight that there is a small risk that peptide administration may induce an inflammatory instead of a tolerogenic response in some circumstances, illustrating the need for incisive understanding of the mechanisms involved in PIT.

#### Late Asthmatic Reactions

PIT has induced late asthmatic reactions (LARs) in some patients during Fel d 1 allergy trials. LARs resulted in reduced lung function occurring at 2-3 hours following treatment and peaking at around 6 hours [62]. LARs were not associated with immediate hypersensitivity reactions/anaphylaxis. It appears that patients with certain HLA types are more likely to experience a LAR, although HLA type alone is not predictive of LAR development [63]. These MHC associations and the timing of LARs point to a T cell mediated mechanism implying that in some patients, cytokines produced by T cells responding to peptide can mediate airway hyperresponsiveness. Crucially, induction of LARs is not required in order to induce T cell tolerance [53] and LARs can be managed by careful regimen planning. In patients who have developed a LAR with PIT, repeating treatment within 2 to 8 weeks using the same dose of peptide induces T cell tolerance without promoting further LARs [53].

#### Challenges of applying PIT to a diverse population, with particular regard to children

##### HLA variation

CD4^+ ^T cells recognise peptide in the context of MHC II and TCR specificity for a peptide is a function of the peptide itself and the MHC II it is presented on. The polygenicity and polymorphic nature of the human leucocyte antigen (HLA) region means that there is extensive variation in the MHC II molecules expressed by different individuals. Most allergens will contain multiple peptide epitopes which can vary in their ability to bind to different MHC II molecules [64], meaning that there will be differences in the allergen-derived peptides which are recognised by different patients' T cells. This poses difficulties for identifying immunodominant peptides capable of inducing tolerance in a HLA-diverse patient population [10]. HLA class II allele associations have been described for allergic diseases [65], yet the complex nature of allergy means that, with the exception of coeliac disease [66], these associations are weaker than for many autoimmune diseases [67]. Although technically possible to characterise immunodominant peptides on an individual patient basis, this approach is unlikely to be feasible both in terms of cost and practicality [68]. But HLA diversity does not preclude the application of PIT to a diverse population [68]. Peptides exhibit considerable promiscuity in their ability to bind to MHC II molecules. A single peptide may bind to MHC II molecules derived from multiple HLA loci [e.g. HLA-DP, -DQ and -DR [69]], and/or to multiple alleles of an HLA molecule [(e.g. HLA-DR1 and HLA-DR53) [70]]. Additionally, use of multiple, overlapping allergen-derived peptides increases the likelihood that a PIT vaccine will contain peptides recognised by a range of HLA-disparate individuals. These characteristics, together with the fact that some HLA types are more common than others, means that a strategic selection of a small number of allergenic peptides could be used to treat a wide range of patients.

##### Nature of the peptide

There are advantages to altering the sequence of a therapeutic peptide. APLs have defined amino acid substitutions which can change MHC and/or TCR binding properties of the peptide [21]. Different alterations can modulate the T cell response elicited by the peptide e.g. APLs that instead of inducing an allergy-associated Th2 T cell response, generate a Th1 response. The latter induces cytokines such as interferon-γ (IFN-γ) which can sometimes antagonise allergic responses [71] and has been found to be expressed by an increased frequency of CD4^+ ^cells in some PIT studies [72]. The stimulatory capacity of a peptide can also be modulated. A "superagonist" APL has an increased ability to stimulate a T cell response whereas an "antagonist" APL can inhibit T cell activation, even in the presence of an agonist. Antagonists have obvious therapeutic applications and have been shown capable of preventing disease in animal models [73,74]. There is concern, however, that an APL defined as an antagonist from its effect *in vitro *on T cells with a limited TCR repertoire may behave differently *in vivo *on encountering a more diverse T cell repertoire [75]. Using APL antagonists therefore risks unpredictable effects, borne out by the aforementioned MS trial [60].

Other applications of APLs could be more amenable to clinical translation. For example, APLs can be generated to reduce the allergenicity of a peptide. *Leech et al *altered a myelin-based peptide reducing its IgG1 binding affinity while maintaining its tolerising capacity [76]. Hence, whilst antagonist APLs may not have widespread application, the potential to alter peptides to improve tolerogenicity or reduce allergenicity should not be dismissed.

##### Route

The most desirable route of delivery of PIT for children would be oral or intranasal as they are minimally distressing. PIT delivered in these ways can be effective in inducing tolerance [32,77], raising hope that these routes may be applicable to the treatment of allergic children in future. It is also possible that a sublingual approach, which has been used to deliver SIT [78], may also prove applicable to PIT. At present however, most allergy-based clinical studies focus on subcutaneous or intradermal PIT administration.

##### Dose and Regimen

There remains no clear consensus as to the optimal peptide dose or regimen which have varied considerably in different trials [49-51,79]. Dose-escalation is advantageous because it is less likely to cause adverse effects and, if side effects such as a LAR develop following a particular dosage, withholding dosage increase until LAR is no longer provoked can be effective [79]. Different regimens may induce different tolerogenic mechanisms. For example, high dose regimens may be more likely to promote a deletional mechanism of tolerance [25] whereas lower dose or multi-dose regimens may provoke induction of IL-10 producing cells capable of regulation [26,35]. Hence, low dose, incremental regimens may be more attractive therapeutically because of the potential for production of regulatory T cells which could exert their effects on pathogenic T cells reactive to other allergens.

A key question is how long PIT needs to be given to induce long-term tolerance? Limited data on this exist from PIT allergy studies which to date have focused on short-term clinical outcomes. However, tolerance following SIT has been demonstrated up to 7 years after completion of treatment [80]. Although these studies involve initial up-dosing followed by maintenance dosing for around 3 years, the frequency and duration of maintenance dosing necessary for long-term tolerance remains undefined. For PIT it is probable that a maintenance regimen will be required to generate long-term tolerance. Ideally such a regimen would be infrequent to encourage compliance, particularly for children. Additionally, maintenance dosage will need to be determined. A high maintenance dose may risk adverse events, particularly if a child has missed a previous dose. Indeed, a review of near fatal reactions to SIT found the majority occurred during maintenance and not during up-dosing [81]. It would be ideal if a low, infrequent maintenance dose was capable of preserving long-term tolerance.

##### Concurrent illness

Concurrent illness may hold particular relevance for application of PIT to children. Data concerning this do not exist for PIT. However, giving SIT during concurrent systemic illness has been associated with fatalities [20] and current guidelines advise against giving SIT during an acute systemic illness [82]. It may be that giving SIT during a period of systemic inflammation can precipitate an inflammatory response and exacerbate disease. It would therefore be advisable to apply these conditions to the use of PIT. There is, however, a lack of data concerning immunotherapy and concurrent viral infection. Children experience a higher frequency of viral infections than adults. Such infections may not cause systemic illness or fever but may still locally activate immune cells such as DCs [83]. Peptide presented to T cells by DCs which have been activated by a viral infection theoretically risks incurring an inflammatory response rather than a tolerant one. Interestingly, in a murine allergic airways model, concurrent influenza infection prevented tolerance induced by intranasal SIT and enhanced allergic inflammation of the airways [84]. In a different study, allergen exposure via the airways at the time of viral infection induced allergen-specific IgG1 and subsequently led to anaphylaxis upon allergen re-exposure [85]. Clearly, these examples may only represent the effects of exposure to whole protein allergen, not peptide, during severe, systemic viral infection. It is thus important to address whether a variety of viral infections, particularly mild non-systemic ones, have any detrimental impact on the outcome of PIT.

## Conclusions

PIT holds promise for the treatment and modification of allergic disease. There have been significant advances in the application of PIT to adult allergic patients, and there is substantial scope for future opportunities for the application of PIT. Yet to maximise therapeutic efficacy necessitates translation to allergic children. For this, further understanding of the underlying mechanisms involved in PIT, potential confounding factors such as viral infection and the interplay between dose, regimen and route need to be further elucidated. Mechanistic studies utilising experimental models together with carefully structured, appropriately powered clinical trials looking at a host of outcome measures should aid future translation to the paediatric clinic.

## List of abbreviations

APL: altered peptide ligand; GATA-3: GATA-binding protein 3; HLA: human leukocyte antigen; LARs: late asthmatic reactions; LPS: lipopolysaccharide; MHC: major histocompatibility complex; PBMC: peripheral blood mononuclear cells; PIT: peptide immunotherapy; SIT: specific immunotherapy; TCR: T cell receptor.

## Competing interests

The authors declare that they have no competing interests.

## Authors' contributions

All 3 authors framed the scope of the article, KJM wrote the article and SMA and JS contributed to refine the substance. All authors read and approved the final manuscript.
